# Co-delivery of VEGF and bFGF via a PLGA nanoparticle-modified BAM for effective contracture inhibition of regenerated bladder tissue in rabbits

**DOI:** 10.1038/srep20784

**Published:** 2016-02-08

**Authors:** Xincheng Jiang, Houwei Lin, Dapeng Jiang, Guofeng Xu, Xiaoliang Fang, Lei He, Maosheng Xu, Bingqiang Tang, Zhiyong Wang, Daxiang Cui, Fang Chen, Hongquan Geng

**Affiliations:** 1 Department of Pediatric Urology, Xinhua Hospital, Shanghai Jiao Tong University School of Medicine, Shanghai, China; 2 Shanghai Institute of Pharmaceutical Industry, Shanghai, China; 3 Key Laboratory for Thin Film and Microfabrication of Ministry of Education, Department of Bio-Nano Science and Engineering, Institute of Micro-Nano Science and Technology, Shanghai Jiao Tong University, Shanghai, China; 4 Department of Urology, Children’s Hospital affiliated with Shanghai Jiao Tong University School of Medicine, Shanghai, China

## Abstract

Graft contracture is a common problem associated with the regeneration processes of tissue-engineered bladders. Currently, most strategies used for incorporating bioactive molecules into biomaterial designs do not work during all phases of tissue regeneration. In this study, we used a growth factor-PLGA nanoparticle thermo-sensitive gel system (i.e., BAM with incorporated VEGF and bFGF-loaded PLGA nanoparticles and mixed with a hydrophilic gel) to promote bladder tissue regeneration in a rabbit model. At 4 and 12 weeks after surgery, contracture rate assessment and histological examination were conducted to evaluate bladder tissue regeneration. The results indicated that the functional composite scaffold continuously and effectively released VEGF and bFGF and promoted bladder reconstruction with a significant decrease in graft contracture. In addition, the number and arrangement of regenerated urothelial cells and smooth muscle cells as well as microvascular density and maturity were improved in the VEGF/bFGF nanoparticle group compared with the single factor VEGF or bFGF nanoparticle group and BAM alone. The nanoparticle thermo-sensitive gel system, which exhibited favourable performance, may effectively inhibit graft contracture and promote bladder tissue regeneration in rabbits.

Varieties of congenital and acquired bladder anomalies and diseases, such as neurogenic bladder, bladder exstrophy, or posterior urethral valves, may require augmentation cystoplasty^1^. The current primary standard of surgical treatment is bladder replacement or augmentation with gastrointestinal segments. However, the substitution of intestinal tissue within the urinary tract is fraught with many potential complications, such as urolithiasis, infection, metabolic abnormalities and even malignancy^2^,^3^. Therefore, urologists have long sought an ideal and optimal substitute to serve as a platform for reliable, complete and functional bladder tissue regeneration.

Bladder acellular matrix (BAM) is an extensively studied scaffold that has been utilized to support bladder augmentation in various animal models and several clinical trials^4^,^5^. Naturally derived BAM retains a porous three-dimensional structure, as well as collagen, elastin, fibronectin and growth factors, which are important for orchestrating the adherence, proliferation, migration and differentiation of multi-layered urothelial cells (UCs), smooth muscle cells (SMCs), endothelial cells and others^6^,^7^. However, the pure degradable scaffold alone does not provide a satisfactory result because it can elicit various negative effects, such as fibroblast depiction, collagen deposition, scar formation, and grafting contracture, over time^8^,^9^,^10^,^11^. Tissues thicker than 0.8 mm or with an area greater than 3 mm^2^ require vascularization to provide cells with adequate nutrients and oxygen and to remove waste and damaged products^12^,^13^. The early and rapid building of a mature vascular network is critical to facilitate grafting of organoids and to support long-term tissue survival^14^. Growth factors are considered to be crucial regulatory molecules throughout the course of tissue regeneration. One strategy to improve the outcome of bladder tissue regeneration is to incorporate a BAM with biologically active growth factors^15^. Many incorporation methods, such as injection, rehydration, and incubation, have been demonstrated to enhance the ability of the BAM to mediate bladder tissue regeneration in the short term (2 or 3 weeks)^16^,^17^,^18^,^19^,^20^,^21^,^22^. However, the introduced growth factors may not have a noticeable effect for a long time. The release pattern of growth factors must correlate with all phases of bladder tissue regeneration.

In our previous study, we have designed a thermal-responsive BAM system containing hydrogel-entrapped protein nanoparticles to promote angiogenesis of the BAM^23^. Instead of directly embedding these bioactive molecules into the BAM, our *in vitro* and *in vivo* studies have demonstrated that this novel system provided a histocompatible environment and acted as an effective drug carrier, exhibiting sustained delivery without an acute inflammatory reaction or toxic manifestation. Furthermore, numerous studies have demonstrated that vascular endothelial growth factor (VEGF) and basic fibroblast growth factor (bFGF) may enhance angiogenesis and smooth muscle regeneration in bladder replacement models^18^,^24^,^25^. In the present study, we used a poly(lactic-co-glycolic acid) (PLGA) nanoparticle (NP)-modified BAM to co-deliver VEGF and bFGF in an effort to rapidly restore vascular networks and to effectively inhibit contracture in augmented bladders. The scaffolds were analysed *in vitro* for protein release. Additionally, the modified scaffolds were implanted in the bladders of rabbits for up to 12 weeks. The implanted scaffolds were analysed for graft contracture, host cell infiltration, vascularization, collagen degradation and deposition, and regenerated smooth muscle strip contractility.

## Results

### Characterization of PLGA NP-modified BAM

**Figure 1:**
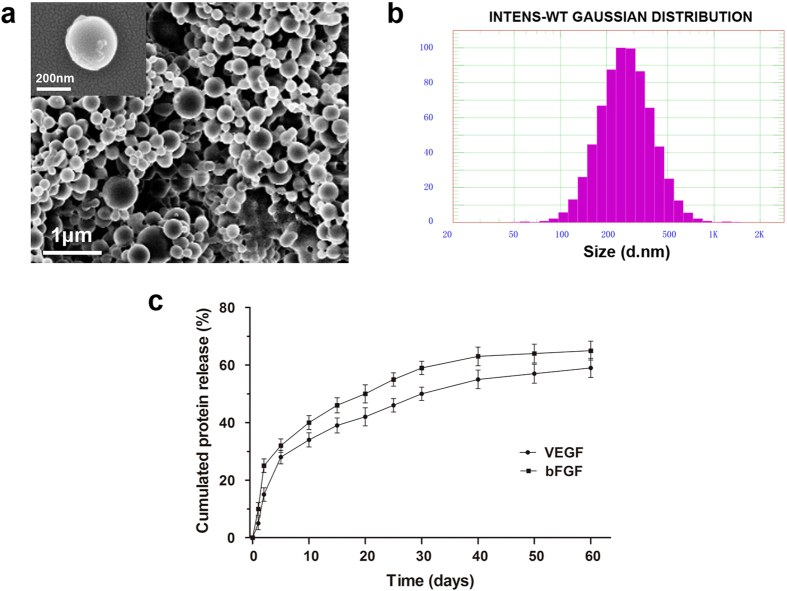
SEM and DLS analyses of protein-loaded PLGA NPs and the *in vitro* release assay. (**a**) Scanning electron microscopic images of VEGF- and bFGF-loaded NPs. (**b**) DLS particle size distributions of VEGF- and bFGF-loaded NPs (Gaussian distribution). (**c**) *In vitro* release assay performed to assess the release profile of the PLGA NPs loaded with VEGF and bFGF.

As shown by scanning electron microscopy (SEM) (Fig. 1a), the VEGF/bFGF-loaded PLGA nanoparticles were observed to be relatively uniform and spherical. These nanoparticles were characterized by dynamic light scattering (DLS), and the average diameter of the NPs was 288.4 ± 15.6 nm (Fig. 1b). The NPs demonstrated a narrow size distribution range between 100 nm and 400 nm, and 90% of the particles were below 600 nm in diameter. The particle size did not change with the addition of VEGF or bFGF. The NPs used in our study exhibited the advantages of being smoothly spherical and having a homogeneous size distribution, which were favourable characteristics for the long-term sustained release of growth factors from the NPs.

Gross observation showed that the BAM was thinner after decellularization compared to the native bladder and retained an intact basement membrane and matrix structure that included collagen, fibronectin, and elastin (see Supplementary Fig. S1a online). Histological analyses of the native bladder and decellularized BAM are shown in Supplementary Fig. S1b. Haematoxylin and eosin (H&E), picric acid-Sirius red, orcein, and 4′,6-diamidino-2-phenylindole (DAPI) staining indicated the intact urothelium, smooth muscle bundles, cellular nuclei, and collagen components in the native bladder. In the BAM, the complete removal of smooth muscle bundles and cellular nuclei was evident, and the extracellular matrix (ECM) structure was maintained. DAPI staining and SEM further demonstrated the complete elimination of residual cells after decellularization.

### *In vitro* release

Figure 1c illustrates the *in vitro* release profiles of VEGF and bFGF from the hydrogel-entrapped nanoparticles in the BAM. Initially, a burst effect was observed during the first 2 days, amounting to 15% and 25% released VEGF and bFGF from the PLGA nanoparticles, respectively. Subsequently, slow and sustained release was observed, and approximately 60% and 70% of the loaded VEGF and bFGF, respectively, were released from the gel embedded-NPs in the BAM over 60 days. The release profiles of the two proteins exhibited similar patterns, demonstrating that the controlled dual delivery of VEGF and bFGF can be achieved by the hydrogel-entrapped drug NP system.

### *In vitro* cell studies

**Figure 2:**
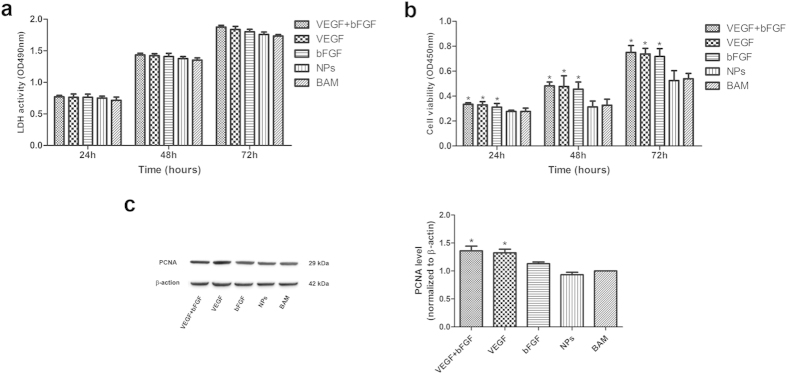
Stimulation of HUVECs by PLGA NPs as assessed by LDH and CCK-8 assays and by western blot analysis. (**a**) LDH analysis of the cytotoxicity of the NP scaffold on HUVECs. (**b**) CCK-8 analysis of *in vitro* cell viability. (**c**) Western blot analysis of the expression levels of PCNA protein (35 kDa) and β-actin (42 kDa) 72 h after drug treatment. The data are expressed as the mean ± SD, and the error bars represent the SD. *p < 0.05 compared with the BAM group.

To investigate the cytotoxicity of the NP scaffold on human umbilical vein endothelial cells (HUVECs), the lactate dehydrogenase (LDH) assay was carried out; the results are shown in Fig. 2a. No significant differences between any of the groups were observed throughout the experiment, indicating that these NPs induced no toxic effects on the cells. Cell viability and proliferation were also examined; these results are shown in Fig. 2b,c. The CCK-8 assay results demonstrated that cells in all the drug-loaded groups (i.e., VEGF + bFGF, VEGF, and bFGF) displayed significantly higher cell viability than did those in the non-factor groups (i.e., NPs and BAM) after 24, 48 and 72 h. According to the western blot results, the expression levels of proliferating cell nuclear antigen (PCNA) protein in the groups treated with VEGF and bFGF were significantly higher than were those in non-factor groups. Altogether, these results indicate that the drug-loaded NP scaffold may be a good candidate bioactive factor delivery system and for bladder tissue engineering applications.

### General observations

All rabbits thrived until the scheduled sampling time, and none of the animals had any complications after the BAM implantation. The surgical incisions healed well, and the graft sites displayed minimal scarring. The non-absorbabl marking sutures were easily distinguishable, and no abnormal symptoms, such as stones, were observed at the luminal surface of the bladders at each time point. By gross observation, the VEGF + bFGF group, the VEGF group and the bFGF group exhibited better phenotypes and therapeutic effects compared with the NP and BAM groups.

### Contracture rate of the BAM

**Figure 3:**
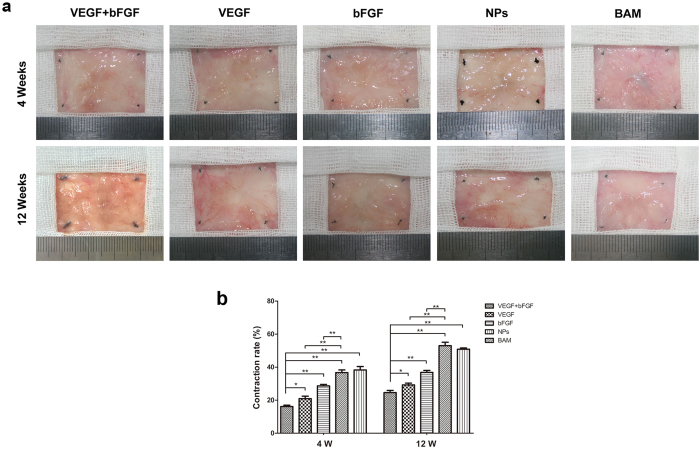
Gross morphological evaluation and contracture rate of regenerated bladder tissues in the five groups. (**a**) Micrographs of the luminal surface of *de novo* bladder tissues removed from the original substitution sites in the five groups at 4 and 12 weeks post-surgery. (**b**) Evaluation of the graft contracture rate. The data are expressed as the mean ± SD, and the error bars represent the SD. *p < 0.05, **p < 0.01.

The contracture rate of the regenerated bladder in each group was determined at 4 and 12 weeks after surgery (Fig. 3a). Compared with the other four groups, the area of regenerated bladder induced by the BAM was larger in the double-factor treated group (VEGF + bFGF), and the contracture rate was significantly lower than that in the control (p < 0.01) (Fig. 3b). At 4 weeks post-implantation, the contracture rate was 20.16 ± 1.77% in the VEGF + bFGF group. At 12 weeks post-implantation, the contracture rate increased to 24.38 ± 1.60%. Compared with the BAM group and NP group, the rate of contracture was significantly lower in the single factor VEGF or bFGF groups (p < 0.01).

### *In vivo* release

The VEGF and bFGF contents of the regenerated bladder were determined using a quantitative protein enzyme-linked immunosorbent assay (ELISA). The amounts of VEGF and bFGF released from the NPs at each time point are shown in Supplementary Fig. S2a and S2b. The results of the *in vivo* drug release analysis demonstrated that the release continued for up to three months. After 12 weeks, the levels of VEGF and bFGF were still higher in the double-factor treated group than in the non-factor-treated groups. The levels of VEGF and bFGF in the groups treated with VEGF and bFGF alone, respectively, were higher than were those in the non-factor-treated groups. These results indicated that our novel drug-loaded PLGA NP-embedded BAM achieved long-term sustained release of growth factors *in vivo*, consistently with the *in vitro* release profiles.

### Histological analysis

**Figure 4:**
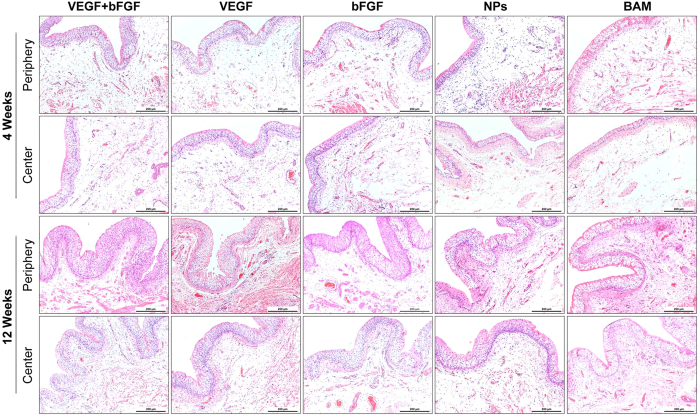
H&E staining of the periphery and centre of the regenerated bladder tissues in the five groups 4 and 12 weeks post-implantation. Scale bars = 200 μm.

Global histological examinations (H&E analyses) at 4 weeks post-operation revealed that a few urothelium layers, smooth muscle bundles, and blood vessels regenerated in all groups and that the peripheral regeneration outweighed the central regeneration (Fig. 4). After approximately 12 weeks, the urothelium had thickened, the number of blood vessels had increased, and the smooth muscle bundles had become larger, all of which resembled native bladder tissue architecture. Through comparison, it was clear that the drug-loaded scaffold groups exhibited massive tissue formation. In particular, the dual drug-loaded scaffold group (i.e., VEGF + bFGF) exhibited the most extensive vascularization and newly formed smooth muscle in both the radial peripheral and central regions of the original implantation sites, demonstrating that it may be the best candidate for bladder tissue regeneration.

**Figure 5:**
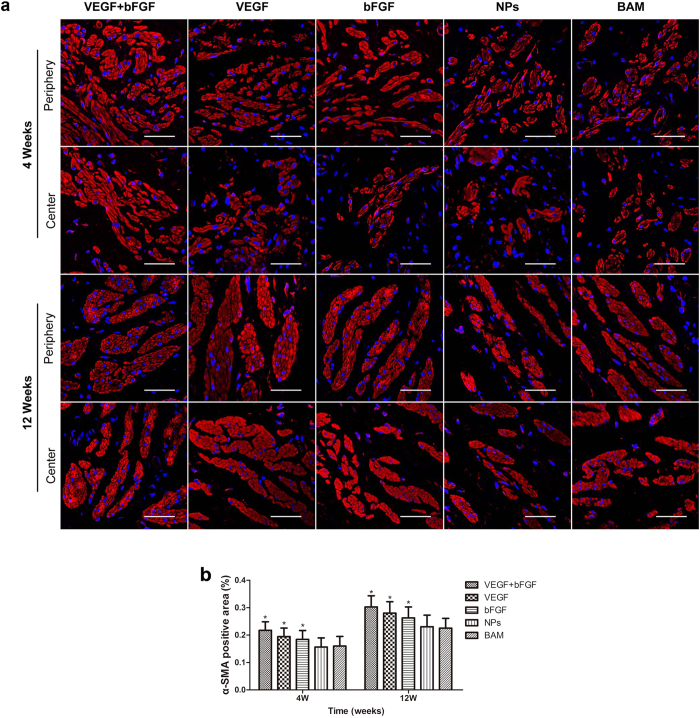
IHC staining for bladder smooth muscle regeneration of the periphery and centre of regenerated bladder tissues in the five groups 4 and 12 weeks post-implantation. (**a**) Micrographs of α-SMA protein expression. For all panels, α-SMA expression is displayed in red, (Alexa Fluor 555) and cell nuclei are displayed in blue (DAPI). Scale bars in all panels = 400 μm. (**b**) Statistical analysis of the percentage of positively stained α-SMA areas. The data are expressed as the mean ± SD, and the error bars represent the SD. *p < 0.05 compared with the BAM group.

Immunohistochemical (IHC) staining was also performed to assess α-smooth muscle actin (α-SMA) expression for the quantification of bladder smooth muscle regeneration. As shown in Fig. 5a, the shape and array of fibrous muscle tissue gradually recovered from 4 to 12 weeks, and the factor-treated groups exhibited much clearer texture than did the non-factor-treated groups. Moreover, statistical analysis revealed that the α-SMA-positive staining area percentage was significantly higher in the factor-treated groups compared to the non-factor-treated groups after 4 and 12 weeks (p < 0.05) (Fig. 5b), indicating that the bioactive factor delivery system showed excellent regeneration induction capability.

**Figure 6:**
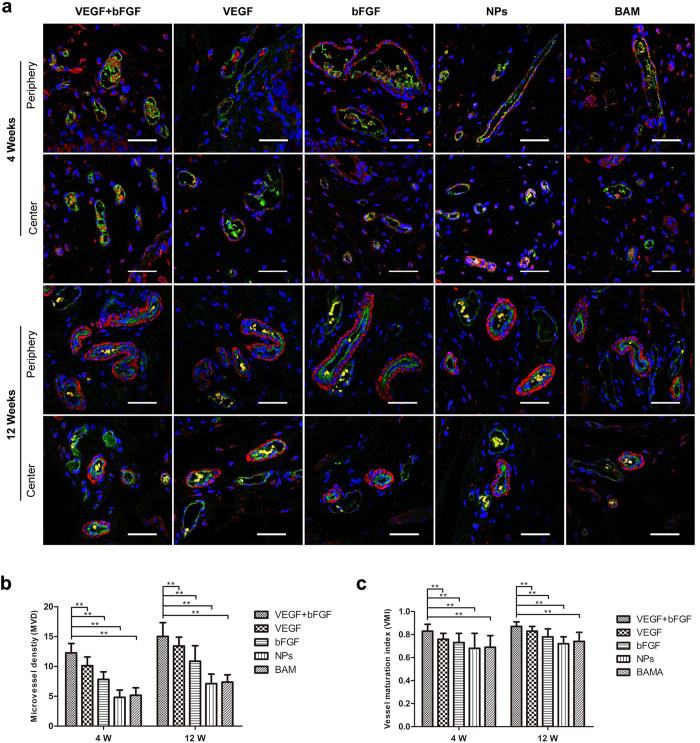
Double-label immunofluorescence combined with CLSM observation of bladder microvascular regeneration of the periphery and centre of regenerated bladder tissues in the five groups at 4 and 12 weeks post-implantation. (**a**) CD31 expression is displayed in green (Alexa Fluor 488), α-SMA expression is displayed in red (Alexa Fluor 555), and cell nuclei are displayed in blue (DAPI). Scale bars in all panels = 400 μm. (**b**) Microvessel density (MVD) and (**c**) Vessel maturity index (VMI) analyses. **p < 0.01 in comparison to the BAM group.

Evidence of *de novo* vascularization processes was observed throughout all regenerated tissues supported by each implant group (Fig. 6a). Vessels containing prominent CD31-positive endothelial cells were found in both the radial periphery and central implantation sites. CD31 and α-SMA double immunostaining was performed to identify and measure the density of new blood vessels and their maturity at 4 and 12 weeks post-implantation. The statistical analysis results are shown in Fig. 6b. The microvessel density (MVD) revealed a considerable number of new capillaries being formed in the VEGF + bFGF group; this formation was significantly superior to that in the other four groups (p < 0.01). The vessel maturity index (VMI) revealed a greater number of mature blood vessels being formed in the double-factor group compared to the other groups (p < 0.01).

**Figure 7:**
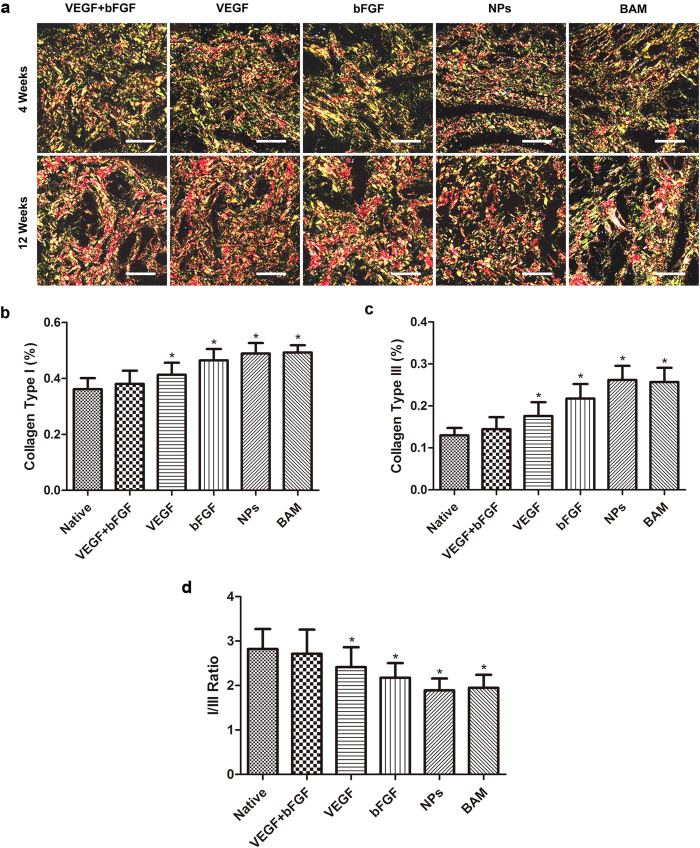
Picric acid-Sirius red staining to assess collagen deposition and degradation. (**a**) Micrographs of collagen protein expression as indicated by picric acid-Sirius red staining. Scale bars in all panels = 100 μm. Analysis of collagen deposition and degradation: (**b**) collagen type I, (**c**) collagen type III, and (**d**) the ratio of collagen type 1 to collagen type III. The data are expressed as the mean ± SD, and the error bars represent the SD. *p < 0.05 in comparison to the native group.

IHC analysis for collagen reflected the deposition and degradation of collagen (Fig. 7a). The content and density of collagen types I and III increased gradually; statistical analysis results that illustrate the increase in collagen are shown in Fig. 7b–d. Significant differences in the contents of collagen types I and III were observed at 4 and 12 weeks among all groups (p < 0.01) (Fig. 7b,c). Significant differences in the ratio of collagen type I to III were also observed at 4 and 12 weeks among all groups (p < 0.05) (Fig. 7d). The percentage of areas positive for collagen types I and III was significantly lower in the VEGF + bFGF group compared to the other four groups. Notably, the ratio of collagen type I to III was higher in the double-factor group than the single-factor and non-factor groups. Although the double-factor-treated group exhibited a higher ratio of collagen I:III, the contents of collagen types I and III were the lowest among all the groups and were much closer to those of the native group.

### The evaluation of isolated strips *in vitro*

**Figure 8:**
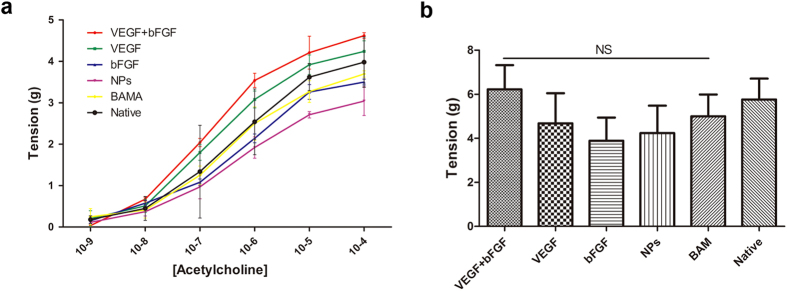
*Ex vivo* contractility evaluations of central regions of both control and regenerated bladder domes supported by scaffold groups at 12 weeks post-implantation. (**a**) Dose response curves for acetylcholine in denuded bladder strips. (**b**) Contractile responses to increased extracellular KCl (120 mM) in the samples detailed in (**a**). The data are expressed as the mean ± SD, and the error bars represent the SD. (NS) p > 0.05 compared with the native group.

Functional evaluation was performed to ascertain the contractile characteristics of bladder muscle strips isolated from both normal and regenerated bladders 3 months after augmentation (Fig. 8). Acetylcholine-induced contractile responses in denuded bladder strips demonstrated similar levels of tensile force in tissues from the growth factor groups (i.e., VEGF + bFGF, VEGF, and bFGF) in comparison to the BAM and NP groups for all steady-state drug concentrations examined (Fig. 8a). The induction of smooth muscle contractility by KCl-mediated membrane depolarization demonstrated similar degrees of tensile force generated by the regenerated tissues from the growth factor groups in comparison to those from the non-factor groups (Fig. 8b).

## Discussion

More recently, a multitude of carrier strategies, e.g., direct absorption, lyophilization, chemical crosslinking and nanoparticle-embedded structures, have been developed^16^,^17^,^18^,^19^,^20^,^21^,^22^. Using these carriers, the release period of bioactive components can be prolonged for a few days, but this extended period still cannot meet the physiological needs of the bladder tissue regeneration process, which usually lasts for weeks or months. Consequently, a long-term carrier is required for the sustained release of bioactive factors. Nanotechnology has emerged as a promising new strategy and has been incorporated into other biomaterials to continuously deliver biologically active molecules for enhanced bladder tissue regeneration^26^,^27^,^28^. In our previous study, we created a nanoparticle thermo-sensitive gel system that was obtained by loading a BAM with thermal gel combined with PLGA nanoparticles incorporated with exogenous growth factors. Thus, our modified graft not only provided a platform that allows regenerated bladder tissue to pass through it but also was capable of providing the long-term sustained release of factors that enhance bladder tissue regeneration. All of the component materials have been approved by the US Food and Drug Administration (FDA) for clinical use. The effectiveness of this system has been demonstrated by our previous studies in rodent models^23^.

In this study, the nanoparticle-embedded gel scaffold was used as a carrier for VEGF and bFGF delivery and was shown to be an effective method to achieve the long-term sustained release of VEGF and bFGF and to maintain the bioactivity of these factors. The results of this study have demonstrated that the modified scaffold exhibited a time-programmed release of the entrapped VEGF and bFGF. The release rate of the nanoparticle thermo-sensitive gel system was slower than that achieved by the current drug-loading methods; this result can be attributed to the slightly increased distance between the nanoparticles and the scaffold caused by the introduction of the thermal gel. Moreover, the determination of the VEGF and bFGF contents *in vivo* was consistent with the *in vitro* release profiles.

Some reports have used a series of indirect urodynamic parameters, such as bladder capacity and bladder compliance, to evaluate graft contracture^17^,^21^,^22^. In our study, we used the preserved graft area to evaluate the contracture rate of the regenerated bladder after augmentation; this evaluation method is more objective and accurate. The results indicated that graft contracture was effectively inhibited to a greater extent under the sustained-release co-stimulation of VEGF and bFGF from the PLGA nanoparticle-modified BAM.

Histological and histochemical examinations were performed to evaluate the bladder tissue regeneration 12 weeks post-surgery. The histochemical analysis indicated that the two double-factor-treated groups showed better regeneration of the urothelium, smooth muscle layers, and blood vessels than did the single-factor groups treated with either VEGF or bFGF. Although VEGF and bFGF use different mechanisms, our results showed that they might act synergistically. Additionally, the non-factor groups showed relatively poor bladder tissue regeneration.

To evaluate the microvascular density and maturity after growth factor stimulation, we chose two indices, MVD and VMI. MVD is the gold standard for evaluating tumour angiogenesis and for estimating prognosis^29^. However, MVD can be used only to evaluate the existence of neovascularization and does not reflect functional status. Therefore, we used VMI for quantitative assessment of the percentage of mature microvasculature positively stained for α-SMA using double-labelling immunohistochemistry with α-SMA- and CD31-specific antibodies in the same tissue slices. The results demonstrated that these two indices of the VEGF + bFGF group were higher than those of the other groups, indicating that VEGF and bFGF-loaded NPs were instrumental in the process of bladder tissue regeneration. This result was also consistent with the histological analysis findings.

Collagen fibres are the principal constituent of the BAM. The content and ratio of collagen significantly affect the mechanical properties of the BAM. The BAM was composed primarily of collagen types I and ΙΙΙ^30^. Although there is no available method to distinguish residual collagen from that of the regenerated tissue, we could evaluate the contents and ratio of collagen types I and III with picric acid-Sirius red staining. The results demonstrated that the contents and ratio of collagen types I and III were more similar to those of normal bladder tissue in the double-factor group than in the other groups. Large amounts of collagen deposition were observed in the non-factor groups, indicating that collagen deposition may be a major cause of scar formation and graft contracture.

Consistently with the results of the histological analysis findings, the *in vitro* isolated strip contractility analysis indicated that simultaneous treatment with VEGF and bFGF post-implantation resulted in the best bladder tissue regeneration among all 5 groups. Our study demonstrated that our engineered functional bladder could be used for bladder tissue regeneration with the desired repair effects in rabbits. Our research may lead to effective therapeutic scaffolds.

Although the nanoparticle thermo-sensitive gel system showed promising results for bladder tissue regeneration, many issues remain that require resolution before the transition to clinical studies. For example, the long-term impact of drug-loaded nanoparticles on bladder function remains to be determined. Therefore, monitoring multiple tumour markers post-implantation is necessary. Understanding the interaction mechanisms between host cells and BAM will be helpful to ameliorate the regeneration process.

## Materials and Methods

### Ethics statement

All animal procedures were approved and supervised by the Institutional Animal Care and Use Committee of the Ethics Committee of Shanghai Jiao Tong University School of Medicine, China and were performed in accordance with the guidelines of the China Act on Welfare and Management of Animals.

### Materials and reagents

Tween® 80 (polyoxyethylene sorbitan monooleate), poly(vinyl alcohol) (PVA; Mw 14–16 kDa) and Pluronic® F127 triblock copolymer were purchased from Sigma-Aldrich (Shanghai, China). PLGA with a 50:50 lactic acid to glycolic acid ratio was purchased from Daigang Biomaterial Co., Ltd. (Jinan, China). Recombinant human proteins (VEGF and bFGF) were purchased from PeproTech (Rocky Hill, NJ, USA). Protein ELISA kits were purchased from Beyotime Biotechnology. All other materials and solvents were of analytical reagent grade.

### Preparation and characterization of PLGA-stabilized VEGF/bFGF NPs

Nanoparticles containing VEGF or bFGF (0.1 μg/mg of NPs) were fabricated by using a previously described double emulsion solvent evaporation technique, with some modifications^31^. In brief, 100 μl of phosphate-buffered saline (PBS) containing VEGF or bFGF was emulsified in 2 mL of dichloromethane containing 20 mg of PLGA using a high-speed IKA Ultra-Turrax homogenizer (IKA, Guangzhou, China) operating at 3,000 rpm for 2 min. Next, the mixture was injected into 4.5 mL of 1.5% PVA and 2% Tween 80 solution to form the double emulsion. The mixture was then re-emulsified at 6000 rpm for 5.5 min over an ice bath and then stirred for at least 3 h at room temperature (RT) to evaporate the dichloromethane completely. The nanoparticles were collected and washed three times with distilled water by centrifugation at 10,000 × g for 5 min at 4 °C; the particles were then freeze-dried at −80 °C for 2 h and vacuum-dried overnight to obtain a dry NP powder.

Particle diameter was measured by DLS using a Zetasizer Nano (PSS Nicomp, Santa Barbara, CA, USA) equipped with a helium-neon laser (5 mW) at a wavelength of 632.8 nm and a scattering angle of 90°. The intensity autocorrelation function was analysed by the cumulate method to obtain the average particle diameter. SEM images were obtained for the assessment of particle shape and surface morphology using a Carl Zeiss SMT (Oberkochen, Germany).

### Preparation of the BAM

The BAM was prepared according to previously published protocols^32^,^33^. In brief, whole bladders harvested from freshly slaughtered pigs were incubated in PBS (pH 7.0) containing 0.1% sodium azide at 4 °C for 24 h. After three washes with ice-cold PBS, the bladders were incubated in 0.5% Triton X-100 (Sigma-Aldrich) for 24 h at RT and then incubated in 0.5% trypsin-EDTA solution for 24 h at 37 °C. The bladder samples were washed three times with ice-cold PBS followed by incubation for 36 h at RT in 1 mol/L NaCl containing 40 U/mL DNase, 40 U/mL DNase I (Sigma-Aldrich) and 40 U/mL RNase A (Sigma-Aldrich). The bladder was then rinsed overnight with ice-cold Tris buffer containing 1% sodium dodecyl sulphate (SDS) and transported in PBS containing 4% sodium deoxycholate and 0.1% sodium azide for 24 h. After the decellularized tissue was washed three times with PBS, it was frozen, lyophilized, sterilized and sealed at −20 °C before use. The native and decellularized bladder tissues were stained with H&E, picric acid-Sirius red, and orcein; the samples were then viewed by scanning electron microscopy (SEM) to detect residual cellular nuclei and ECM components.

### Release *in vitro*

The release of VEGF or bFGF was measured in a buffer solution using a PLGA NP-embedded thermo-sensitive hydrogel as described previously^23^. In brief, 10 mg of lyophilized VEGF or bFGF-loaded NPs was resuspended in 25% Pluronic® F127 solution and then rapidly embedded into the BAM with a multipoint sequential injection system. The matrix was placed in 2 mL of PBS (pH 7.4) containing 0.02% w/v sodium azide and placed in a thermostatic incubator shaker at 100 rpm and 37 °C. At predetermined intervals, 5 mL of release medium was removed and replaced with 5 mL of fresh, filtered buffer, which had been centrifuged for 10 min at 20,000 × g and 4 °C. The cumulative VEGF or bFGF percentage released in the supernatant was determined using ELISA. All the operations were analysed in triplicate.

### *In vitro* cell experiments

HUVECs (Chinese Academy of Sciences, Shanghai, China) were used in the study. The cells were propagated in Dulbecco’s modified Eagle’s medium (DMEM, HyClone) supplemented with 10% foetal bovine serum (FBS, Gibco) and maintained at 37 °C with 5% carbon dioxide. The cell culture medium was changed every 3 days.

For the *in vitro* assay of cell viability and assessment of the cytotoxicity of the drug-loaded PLGA NPs, cells were seeded in 96-well plates at a density of 1 × 10^3^ cells/well and allowed to adhere for 24 h. The medium was then replaced with serum-free medium supplemented with (or without) different NPs with final concentrations of 10 ng/mL VEGF and 10 ng/mL bFGF. The treated cells were incubated for another 24, 48 and 72 h. The LDH analysis was performed using an LDH cytotoxicity assay kit according to the manufacturer’s instructions (Beyotime, Shanghai, China). Cell viability was assessed by CCK-8 assay according to the manufacturer’s instructions (Dojindo, Japan).

For the western blot analysis, HUVECs were seeded in 6-well plates at an initial density of 2 × 10^4^ cells/well. As described above, the cells were treated with different drug-loaded PLGA NPs. After the cells were incubated for another three days, the total cellular protein was extracted, and the protein concentrations were determined by bicinchoninic acid assay (Beyotime, Shanghai, China). Protein samples were separated by SDS-PAGE, transferred to PVDF membranes (Millipore), and blocked in 5% non-fat milk. The membranes were then incubated with anti-PCNA (1:2000, ab92552, Abcam) or anti-β-actin (1:1000, Jackson ImmunoResearch Laboratories) diluted in primary antibody dilution buffer at 4 °C overnight. Subsequently, the membranes were washed and incubated with secondary HRP-conjugated anti-rabbit antibody (1:20000; Abcam) or anti-mouse antibody (1:10000; Jackson ImmunoResearch Laboratories) in 5% non-fat milk for 2 h. For densitometric quantification, the signal intensities were visualized and quantified using the Bio-Rad Chemi Doc^TM^ XRS+ system with Image Lab^TM^ Software.

### Animal augmentation cystoplasty

Fifty male New Zealand white rabbits weighing 2.0–3.0 kg were obtained from the Animal Centre of Shanghai Jiao Tong University School of Medicine, China. The rabbits were randomly divided into five groups, namely, VEGF + bFGF, VEGF, bFGF, NPs alone and BAM alone, and kept in separate cages at room temperature (20–24 °C) under a 12 h light/dark cycle. The rabbits were fasted overnight before surgery. The animals received general anaesthesia with 3% pentobarbital (1 mL/kg, ear vein) and were then placed on the operating table in a supine position. The suprapubic region was disinfected with a povidone iodine scrub, and the abdomen was shaved and washed. Respiration and heart rates were monitored continuously throughout the period of anaesthesia. The abdomen was opened through a lower midline incision, and the bladder was exposed. After the bladder was emptied, it was filled with 20 mL of sterile saline solution. The dome of the bladder was incised longitudinally, and a 2 × 3 cm scaffold was anastomosed to the bladder defect using 5–0 absorbable polydioxanone sulphate (PDS) sutures in a running locking stitch. Marking sutures were placed at all 4 corners of the scaffold with 3–0 polypropylene non-absorbable suture material. Organ distension was performed with saline infusion following scaffold integration to ensure that no gross leaks were present through the scaffold centre or along the suture line. Rectus abdominis fascia and skin incisions were subsequently closed with running sutures. All animals were kept on prophylactic antibiotics for 7 d post-operation. Five rabbits in each group were euthanized at 4 and 12 weeks post-implantation, and isolated bladder specimens were subjected to *in vivo* release, histological, IHC, and mechanical analyses.

### Quantification of proteins in the BAM

The *in vivo* release amounts of VEGF and bFGF in the matrix at each time point were measured by extracting and quantifying regenerated bladder harvested from the original defect site. In brief, harvested regenerated tissue segments were weighed and placed into a cell lysis and protein solubilization buffer (RIPA, Sigma-Aldrich) with a protease inhibitor (PMSF, 1:100, Sigma-Aldrich). The samples were then processed using sonication followed by centrifugation. The supernatant was collected, and total levels of VEGF and bFGF were determined using ELISA according to the manufacturer’s instructions. All experiments were performed in triplicate.

### Contracture rate of the BAM

The contracture rate of grafts in the five groups was evaluated at 4 and 12 weeks after augmentation. In brief, the whole bladder was retrieved, and the internal and external surfaces of the relaxed bladder were observed. The graft was photographed using a digital camera, and the graft size was defined by measuring the distances between the non-absorbable sutures and then analysed using Image-Pro Plus 6.0 software. The contracture rate of the graft was calculated according to the following equation: (the area of original graft - the area of regenerated bladder) / the area of original graft ×100%.

### Histological and IHC analyses

Regenerated bladder segments from both the periphery and centre of the substitution site were obtained for histological analyses at 4 and 12 weeks post-surgery. In brief, the specimens were fixed in 4% paraformaldehyde solution, dehydrated in xylol and graded alcohols, and then embedded in paraffin wax. Sections (5-μm thick) were prepared and stained with H&E.

For the IHC analyses, α-SMA and CD31 were detected using double-label immunofluorescence staining. As in the procedures described above, five-micrometre sections were rehydrated and treated with antigen retrieval solution. Triton X-100 (0.2%) was added, and the sections were incubated for 5 min. The samples were then blocked with 5% donkey serum in PBS for 30 min. The sections were incubated with mouse anti-rabbit CD31 antibody (Abcam, 1:20) together with goat anti-rabbit α-SMA antibody (Abcam, 1:200) overnight at 4 °C. The sections were then incubated with the secondary antibodies Alexa Fluor 488 donkey anti-mouse (Invitrogen 1:200) and Alexa Fluor 555 donkey anti-goat (Santa Cruz, 1:200) for 1 h at 37 °C. After the sections were counterstained with a DAPI solution (Beyotime Biotechnology), the sections were mounted with Fluorescent Mounting Medium (Dako). Microscopic observation and photography were performed using a confocal laser scanning microscope (CLSM, Zeiss Confocal LSM 710 microscope, Carl Zeiss). MVD was measured according to the method described by Weidner *et al.*^34^. The sections were scanned at low power (magnification × 100) to identity three hot spots of the highest neovascularization, and then individual microvessel counts were made using a 200× field. The percentage of mature blood vessels in each sample was calculated based on the following formula: VMI = (total No. of α-SMA-positive blood vessels / total No. of CD31-positive blood vessels) × 100%. All blood vessels were numbered and determined to be positive or negative for CD31 and α-SMA expression by two trained and reliable investigators.

To assess the deposition and degradation of collagen types I and III, regenerated tissue sections (5 μm thickness were stained with Picric acid - Sirius red (0.1% Sirius red in saturated aqueous Picric acid) (Sigma, USA) for examination of collagen types I and III. Stained sections were scanned under polarizing microscope (Olympus BX51). The images of three randomly selected areas per section were digital captured (magnification × 100) and the optical density of collagen types I and III was analysed using the hue saturation intensity (HSI) colour model in the Image-Pro Plus 6.0 image software. The percentage of positive area was used to express the content and the ratio of collagen types I and III in the regenerated tissues.

### *Ex vivo* contractility of Isolated Strips

At 12 weeks post-implantation, full-thickness regenerated bladder strips from each group were obtained from the centre of the original repaired site; in addition, native strips were extracted from similar regions of the bladder that had not been subjected to surgery to perform the *ex vivo* contractility analyses as previously described^9^,^35^. In brief, bladder tissue was carefully sectioned into 1.5 × 0.5 cm strips that were suspended in an organ bath with 10 mL of Krebs’ buffer at 37 °C and bubbled with 95% O_2_ and 5% CO_2_. Tissues were linked with an isometric force displacement transducer at a preload of 1.5 g, and then they were equilibrated for 60 min before drug testing. Contractile responses to a concentration gradient of acetylcholine (cholinergic agonist, 10^−9^–10^−4^ mol/L) and KCl (120 mM) were measured. The transducer signals were continuously obtained and recorded by a multi-channel physiological signal acquisition system.

### Statistical analysis

All data are expressed as the mean ± standard deviation (SD). Statistical analyses were conducted using SPSS statistics software v19.0. The bladder tissue regeneration assays were assessed by one-way analysis of variance (ANOVA), whereas multiple comparisons between two groups were determined by the LSD and Student–Newman–Keuls (SNK) multiple comparison test. Statistically significant values were defined by *p < 0.05 and **p < 0.01.

## Additional Information

**How to cite this article**: Jiang, X. *et al.* Co-delivery of VEGF and bFGF via a PLGA nanoparticle-modified BAM for effective contracture inhibition of regenerated bladder tissue in rabbits. *Sci. Rep.* **6**, 20784; doi: 10.1038/srep20784 (2016).

## Supplementary Material

Supplementary Information
