# Supplementary material for: Co-delivery of VEGF and bFGF via a PLGA nanoparticle-modified BAM for effective contracture inhibition of regenerated bladder tissue in rabbits
Source: Sci Rep. 2016 Feb 8;6:20784. doi: 10.1038/srep20784 (PMC4745101; doi:10.1038/srep20784)
Supplement: Supplementary Information [file srep20784-s1.pdf]

Supplementary Information

Co-delivery of VEGF and bFGF via a PLGA nanoparticle-modified BAM for effective contracture inhibition of regenerated bladder tissue in rabbits

Xincheng Jiang [1, †], Houwei Lin [1, †], Dapeng Jiang [1], Guofeng Xu [1], Xiaoliang Fang [1], Lei He [1], Maosheng Xu [1], Bingqiang Tang [1], Zhiyong Wang [2], Daxiang Cui [3], Fang Chen [4], Hongquan Geng [1, *]

[1] Department of Pediatric Urology, Xinhua Hospital, Shanghai Jiao Tong University School of Medicine, Shanghai, China

[2] Shanghai Institute of Pharmaceutical Industry, Shanghai, China

[3] Key Laboratory for Thin Film and Microfabrication of Ministry of Education, Department of Bio-Nano Science and Engineering, Institute of Micro-Nano Science and Technology, Shanghai Jiao Tong University, Shanghai, China

[4] Department of Urology, Children's Hospital affiliated with Shanghai Jiao Tong University School of Medicine, Shanghai, China

* Corresponding author. Department of Pediatric Urology, Xinhua Hospital, Shanghai Jiao Tong University School of Medicine, 1665 Kongjiang Road, Shanghai, 200092, China.
E-mail address: ghongquan@hotmail.com (H. Geng)

[†] These authors contributed equally to this work.

Supplementary Figure S1

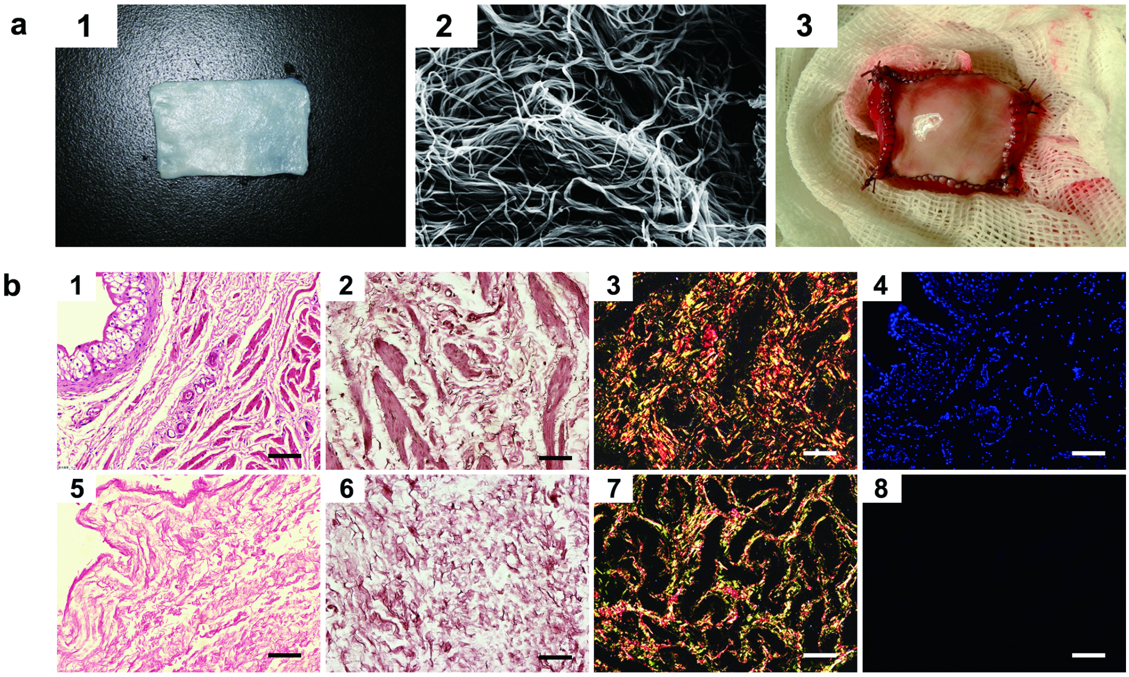

The appearance of the porcine BAM. In graph (a), 1 is a photograph of BAM, 2 is an SEM image of BAM, and 3 depicts rabbit bladder augmentation cystoplasty with the matrix groups. In graph (b), 1 and 5 show H&E staining of the native bladder tissue and BAM (scale bars = 100 μm), 2 and 6 show orcein staining of the native bladder tissue and BAM (scale bars = 50 μm), 3 and 7 show picric acid-Sirius red staining of the native bladder tissue and BAM (scale bars = 100 μm), and 4 and 8 show DAPI staining of the native bladder tissue and BAM (scale bars = 200 μm).

Supplementary Figure S2

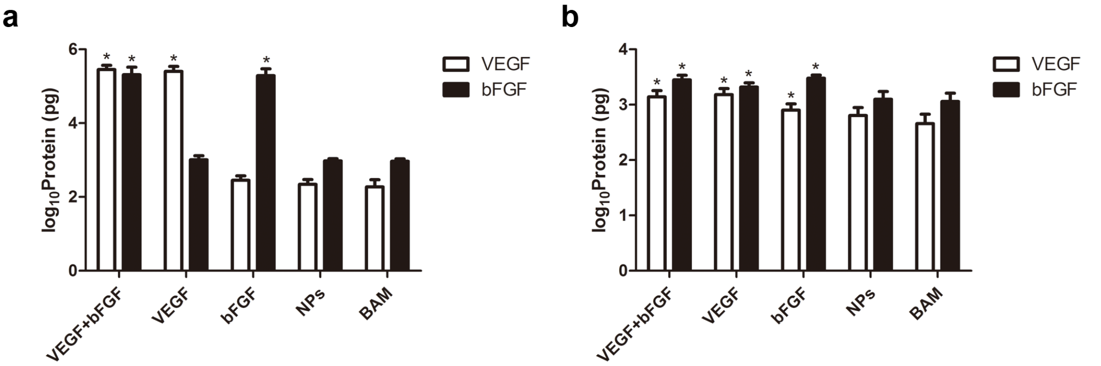

The *in vivo* release of VEGF and bFGF in the BAM. (a) The amounts of VEGF and bFGF released from the NPs at 4 weeks. (b) The amounts of VEGF and bFGF released from the NPs at 12 weeks. The data are expressed as the mean ± SD, and the error bars represent the SD. (*) $p < 0.05$ compared with the BAM group.
